# GxGrare: gene-gene interaction analysis method for rare variants from high-throughput sequencing data

**DOI:** 10.1186/s12918-018-0543-4

**Published:** 2018-03-19

**Authors:** Minseok Kwon, Sangseob Leem, Joon Yoon, Taesung Park

**Affiliations:** 1 Department of Biomedical Informatics, Harvard Medical School, Boston, 02115 MA USA; 2 Department of Statistics, Seoul National University, Seoul, 08826 South Korea; 3 Interdisciplinary program, Seoul National University, Seoul, 08826 South Korea

**Keywords:** Gene-gene interaction, Rare variant, Multifactor dimensionality reduction

## Abstract

**Background:**

With the rapid advancement of array-based genotyping techniques, genome-wide association studies (GWAS) have successfully identified common genetic variants associated with common complex diseases. However, it has been shown that only a small proportion of the genetic etiology of complex diseases could be explained by the genetic factors identified from GWAS. This missing heritability could possibly be explained by gene-gene interaction (epistasis) and rare variants. There has been an exponential growth of gene-gene interaction analysis for common variants in terms of methodological developments and practical applications. Also, the recent advancement of high-throughput sequencing technologies makes it possible to conduct rare variant analysis. However, little progress has been made in gene-gene interaction analysis for rare variants.

**Results:**

Here, we propose GxGrare which is a new gene-gene interaction method for the rare variants in the framework of the multifactor dimensionality reduction (MDR) analysis. The proposed method consists of three steps; 1) collapsing the rare variants, 2) MDR analysis for the collapsed rare variants, and 3) detect top candidate interaction pairs. GxGrare can be used for the detection of not only gene-gene interactions, but also interactions within a single gene. The proposed method is illustrated with 1080 whole exome sequencing data of the Korean population in order to identify causal gene-gene interaction for rare variants for type 2 diabetes.

**Conclusion:**

The proposed GxGrare performs well for gene-gene interaction detection with collapsing of rare variants. GxGrare is available at http://bibs.snu.ac.kr/software/gxgrare which contains simulation data and documentation. Supported operating systems include Linux and OS X.

**Electronic supplementary material:**

The online version of this article (10.1186/s12918-018-0543-4) contains supplementary material, which is available to authorized users.

## Background

Looking beyond single genetic effects and the boundaries of additive inheritance of single nucleotide polymorphisms (SNPs), could better demonstrate biological pathways involved in disease etiology [1]. Many assumed common variants to provide sufficient explanation for common diseases, so the focus of genome-wide association studies (GWAS) have been set on evaluating common genetic markers. Such approach is also known as the ‘common-disease common-variants’ model, which states that common diseases are caused by common variants with minor allele frequencies (MAFs) greater than 5% [2]. Although the success of GWAS in common diseases provided some convincing evidences, a large proportion of the genetic heritability is left unresolved using the currently discovered major genetic loci [3]. For instance, the result with 71 loci of a genome-wide meta-analysis explains only 23.2% of the heritability of Crohn’s disease [4]. Such shortcomings led to a phase of analyzing the rare variants, genetic interactions, and environmental interactions. Hence, more complex statistical methods had to be applied to detect genes with not only moderate or high effect size, but also small marginal effect towards phenotype that interacts with each other.

In recent years, studies support the ‘common-disease rare-variants’ hypothesis [5], which claims that complex disorders are caused by multiple rare variants. As an example, type 2 diabetes mellitus (T2D) is a complex disease which is caused by both genetic composition and environmental factors. The exact biochemical mechanism is yet to be unveiled, however, though impairments in insulin action and secretion certainly take parts. Unlike type 1 diabetes, T2D is characterized primarily by ‘insulin resistance’, and a vast majority of this resistance is shown as defects at the post receptor level [6]. Heterogeneity in pathological and physiological symptoms of T2D leads to a variety of complications such as coronary heart disease, retinopathy, nephropathy, etc.

Due to rare variants having low frequencies and existing in large number, traditional single-marker association tests generally lack power in these variants. In recent studies, several methods have been developed and categorized to one of collapsing, similarity-based and distance-based methods. Collapsing methods transform multiple variants in specific regions of interest to a single new aggregated variable. Combined Multivariate and Collapsing (CMC) method [7], Weighted Sum (WS) test [8], Variable Threshold (VT) [9] and GRANVIL [10] belong to this category and they show better performance than others when variants in a region have effects in the same direction (deleterious or protective) and when there are a few non-causal variants in the region. Similarity-based methods use a multi-locus genotypes similarity among samples. Kernel-based adaptive cluster method [11] and sequencing kernel-based association test (SKAT) [12] belong to this category, and these methods are robust to directions of variant effects and large proportion of non-causal variants in a region. SKAT is extended to SKAT-O [13] using weighted average of statistics of SKAT and burden test. Distance-based methods use physical positions of variants. IL-K [14], KERNEL [15] and CLUSTER [16] belong to this category.

However, studies on gene-gene interaction (GGI) studies using rare variants are scarce. We incorporate the multifactor dimensionality reduction (MDR) method, which is useful for “detecting and characterizing interactions in common complex multifactorial disease” [17]. This is applicable even when the sample size is small or when the dataset contains alleles in linkage disequilibrium. However, the original MDR method had some limitations, and various versions of improved methods were suggested, such as the Odds ratio based MDR [18], Log-linear model-based MDR [19], gene-based MDR [20], entropy-based GGI analysis algorithm (IGENT) [21], etc. Of these methods, a generalized version of MDR called GMDR [22], enables the use of covariates and continuous phenotypes as the dependent variable. Its basic idea is to substitute a score statistic or some other quantitative measure, instead of disease status, while preserving the same reduction strategy.

To apply MDR in rare variant setting, we proposed GxGrare that applied collapsing strategies in order to summarize the genotype information to a more practical score that can be used in the existing MDR methods. We then compared the performances of our method with that of the Summation of Partition Approach (SPA) [23]. The SPA method has been proposed by Fan et al. and it is a robust model-free method that is designed to detect both marginal effects and effects due to gene-gene and gene-environment interactions of rare variants. The SPA method is one of the latest approaches, yet it defines gene-gene interaction in a somewhat different manner in that it measures the interaction between two SNPs within a gene. Our simulation scheme includes those from this paper.

## Methods

In the methods section, we introduce a novel collapsing idea. Then, we briefly review the GMDR method and evaluation measures. Last, we explain our proposed analysis steps.

### Collapsing methods

**Fig. 1:**
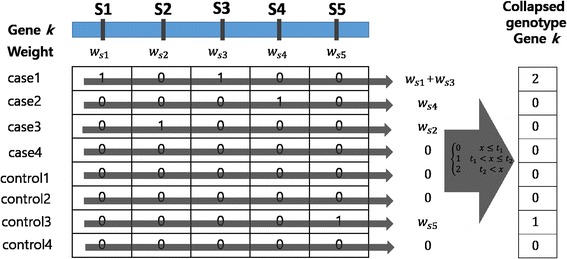
Illustration of the collapsing strategy shared in all three proposed methods

Since rare variants are hard to analyze by traditional single-marker association methods, we propose a novel collapsing method. In our collapsing method, the genotypes of SNPs in a gene are merged with a new genotype of the gene with the SNPs’ weights, and we propose novel three weighting schemes for individual SNPs: MAF-based, functional region-based, and effect-based collapsing. The common collapsing strategy is shown in Fig. 1.

Figure 1 shows an illustrative example that consists of 4 case and 4 control samples. In the gene *k*, there are five SNPs (S1~S5) and their weight values (w_s1_~w_s5_). Each sample has five genotypes that represent the number of mutations in a SNP. First, we calculate the weight of each SNP by one of the three proposed weighting schemes. Second, we multiply genotype values and corresponding weights and then sum them for each sample such as ‘w_s1_ + w_s3_’ for case1. Last, summed values are transformed to one of two or three values by a binning step (gray arrow). The number of bins and threshold values for the binning step depend on the weighting scheme.

In MAF-based collapsing, the weight of SNPs inside the genes are decided based on their MAFs; MAF < 0.01 (weight: 1) and the rest (weight: 0). This weighting scheme is designed based on the hypothesis that “disease-promoting variants should be rare” [24]. We tested two kinds of number of bins, two and three. In the two bins test, the collapsed genotypes are decided by whether summed values are zero (collapsed genotype: 0) or not (collapsed genotype: 1). In other words, genes are divided into two with (collapsed genotype: 1) or without (collapsed genotype: 0) rare variants groups. In the three bins test, genes are divided into three groups: genes without rare variants, genes with a single rare variant and genes with two or more rare variants groups.

For the functional region-based collapsing, the variants in a gene are collapsed to their annotated functional regions, such as coding region, splice junctions, etc. Genes with rare variants in a less functional region, such as intron region, have low weight such as 0, and 1 otherwise. For the simplicity of weighting scheme, we use two values 0 or 1 as weight values in our tests. In other words, all variants would be given the same weight, 1, and a variant in functional region would be given its SNP count through an indicator function, less meaningful variants would be given 0. The sum of the output SNP counts of the indicator function would be compared to a threshold of 0. This reflects the importance of gene structure and region specific variants. The information of functional region can be derived from annotation scores of SNPeff [25], PolyPhen2 [26] and SIFT [27]. However, scores of PolyPhen2 and SIFT have poor coverage (only 60 and 81% of human proteome, respectively). Therefore, a conservation score of variants can be an alternative weight as functional region information additionally, under the claims of Ng and Henikoff, authors of SIFT [27], that “disease-causing mutations are more likely to occur at positions that are conserved throughout evolution.” The conservation scores calculated from PhastCons [28] and phyloP [29] are utilized. Among various candidates of functional region information, we used the regional annotation tag information of SNPeff that variants are pooled into four categories highly deleterious, moderately deleterious, less deleterious and others. We set weights of variants as 1 in highly and moderately categories or 0 for the other variants.

**Fig. 2:**
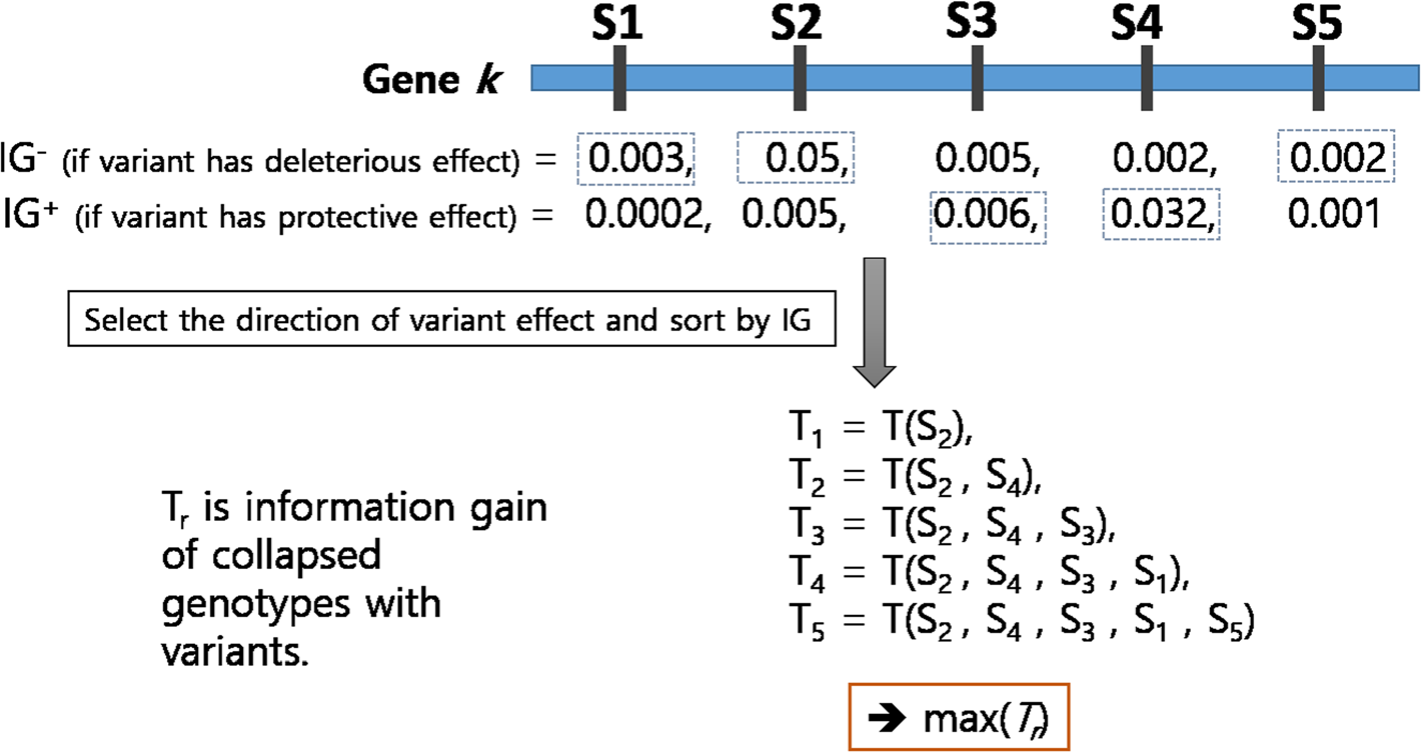
Illustration of the effect-based collapsing scheme

Aforementioned two weighting schemes are based on the unidirectional mechanism that mutations may increase disease risks. However, even if it has a low chance of happening, mutations can have protective effects at diseases occurrences. Therefore, we proposed the effect-based scheme based on a bidirectional (deleterious and protective effects) mechanism. The effect-based method collapses the variants to their according genes based on values of information gain (IG) under assumptions of both effect direction (deleterious and protective). IG value is one of the association measures based on information theory and we elaborated IG in the following 2.3 Evaluation Measures section. First, two IG values of a variant are calculated for deleterious and protective effect as shown in Fig. 2. These two IG values are calculated by number of mutations in the case group and control group respectively. Second, variants are selected sequentially by order of IG values similar to the forward selection approach and IG value of selected variants combination is calculated. The different number of variants (T_1_~T_5_ in Fig. 2) are compared by their IG values and the number of variants is determined the maximum IG value.

### Review of generalized multifactor dimensionality reduction (GMDR)

MDR method is proposed for gene-gene interaction detection in case-control study. The key idea of MDR is the reduction method from multi-locus genotypes into one of high or low risk groups. Since MDR is a non-parametric approach, the best SNP combination is selected by evaluation measures such as balanced accuracy (BA) and cross validation consistency (CVC) in the cross validation procedure. Among many extensions of MDR, GMDR is extended for covariate adjustments and to deal with not only quantitative phenotypes such as a body mass index (BMI) but also qualitative phenotypes such as disease status.

**Fig. 3:**
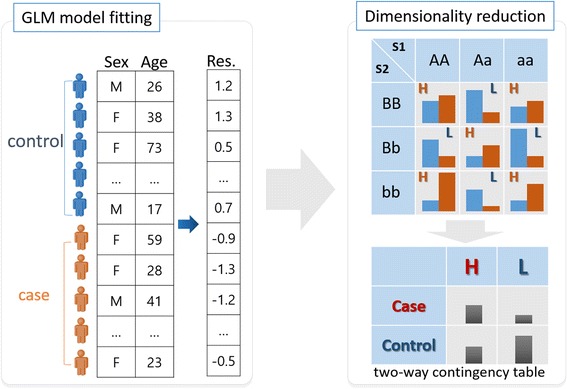
An example of GMDR

Since the purpose of GMDR is to detect interactions of SNP combinations with covariate adjustments for both quantitative and qualitative phenotypes, it consists of two steps. In the first step, residuals of each sample are calculated using a model fitting of the generalized linear model (GLM) with covariates and without genotype information. In the second step, residuals are used as genotypes in MDR. For an illustrative purpose, an example of GMDR is shown Fig. 3. In Fig. 3, two covariates (sex and age) are considered in a case-control study. The first step is a GLM fitting in Fig. 3 and the residual values are calculated by the fitted model. And then, the residual values are pooled into groups by genotype values of a SNP combination as shown in Fig. 3. In this example, two SNPs (S1 and S2) make the SNP combination and ‘A’ designates major allele of the S1, ‘a’ designates minor allele of the S1, ‘B’ designates major allele of S2 and ‘b’ designates minor allele of S2. In this step, residuals are grouped by own sign and summed respectively in each genotype combination. In Fig. 3, the left blue bars represent the sum of plus signed residuals and the right orange bars represents the sum of minus signed residuals in each genotype combination. And then, each cell is assigned a high (H) or low (L) risk group by a comparison of heights between the blue bar and the orange bar. These two steps (GLM model fitting and dimensionality reduction) are performed for each SNP combination and compared by evaluation measures under the cross-validation structure. For example, in 10-fold cross-validation, data is divided into 10 subsets. A GMDR model is trained using 9/10 among subsets, and then the GMDR model is tested to remained 1/10 subset.

We use collapsed genotypes of genes as genotypes of SNPs in GMDR method. Since, a collapsed genotype of a gene has two (0/1) or three (0/1/2) possible values, the size of a contingency table in the dimensionality reduction step, can be the same or reduced, but the final two-way contingency table has the same size.

### Evaluation measures

Since the results of GMDR method, as other methods, depend on evaluation measures, different variants combinations can be selected sometimes by selection of evaluation measures. Therefore, we concerned two popular MDR evaluation measures BA and CVC, and an information theory based measure IG for case-control studies.

Many extensions of MDR for case-control studies use BA and CVC as evaluation measures. BA defined as an average of sensitivity and specificity, is proposed by Velez et al. to measure the performance in MDR methods [30]. IG, also known as Kullback-Leibler divergence, proposed by Mitchell et al., is defined as *IG*(*T*, *g*) = *H*(*T*) − *H*(*T*| *g*), (where *H* is entropy). In genetic association studies, *T* can be a phenotype such as disease status and g can be a genetic variant such as a SNP. In this case, entropy of *T H*(*T*) represents the uncertainty of *T* (*H*(*T*) is 1 on a balance case-control study.) and *H*(*T*| *g*) represents remained uncertainty under known *g*. Therefore, *IG*(*T*, *g*) represents the amount of uncertainty reduction of *T* by *g*. IG is sometimes called mutual information and it corresponds to a gamma distribution asymptotically [31]. *IG*(*T*, *g*) holds the meaning of difference between marginal and conditional entropy and is from information theory and machine learning that can be used as an evaluation measure. In a dataset, with given disease information and collapsed genotype information, the conditional and marginal entropy can be easily calculated.

CVC, along with the BA, has been used multiple times in MDR studies; it is defined as the number of times that a SNP combination is identified as the best combination, from any measures (i.e. BA or IG), across the 10 CV datasets. A general step of 10-fold CV is done by splitting the full dataset into 10, and using 1/10 as testing set and others as training set. This way, ten different training sets and testing sets can be analyzed. We use weighted CVC (WCVC) [32] in T2D data analysis to decide the optimal order interactions for advanced analysis.

### Proposed GGI analysis method for rare variants (GxGrare)

**Fig. 4:**
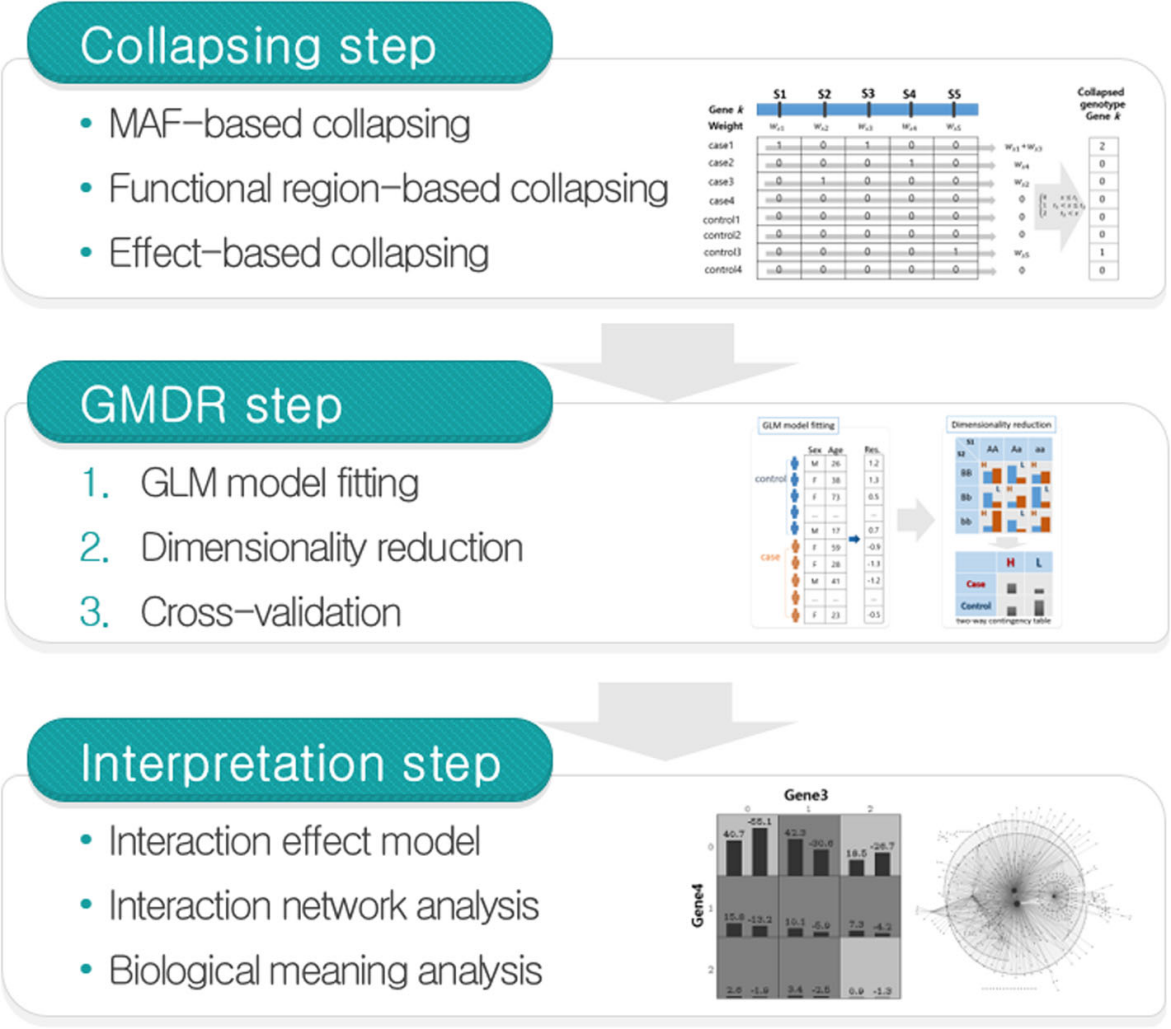
Three steps in the proposed method

Figure 4 explains the steps involved in the proposed method. In the collapsing step, one among three collapsing schemes is selected, and then information of variants on a gene are aggregated to a new collapse genotype under the selected collapsing scheme. We tested three collapsing schemes and compared the results.

In the GMDR step, first, residuals of samples are calculated through GLM model fitting with covariates. Second, H or L value is assigned for each combination of collapsed genotype by a distribution of residuals and then dimensionality is reduced by MDR manner. The best SNP combinations are selected by the evaluation measures under the cross-validation structure.

The selected SNP combinations in the results of GMDR step, are analyzed in the interpretation step. Basically, the interaction of the selected SNP combination is represented through visualization. For an advanced analysis, interactions can be shown as a network diagram. Biological meaning of SNP combinations and its interaction may be confirmed or interpreted by literature reviews. More details of interpretation steps are shown in the result section.

## Results

First, we compared our proposed method using optional selections (collapsing schemes and evaluation measures) with the summation of partition approach (SPA) in terms of power to detect causal variants under various simulation settings. Second, we checked type I error rates of our proposed method and SPA using a simulation dataset without causal variants. Then, we applied the proposed method with MAF-based collapsing to T2D data to detect interactions using rare variants and interpreted the results.

### Results of simulation

The statistical efficiency of the proposed method was evaluated through a set of gene-gene interaction simulation settings. We incorporated Marchini’s four interaction models for rare variants: multiplicative, additive, maximum and minimum threshold effects models [33]. The genotypes were generated under HWE with 20 rare SNPs in each gene. As for the phenotypes, 2 cases were considered; GGI with and without marginal effects. Also, to handle the directions of SNP effects, deleterious vs. protective, we considered unidirectional and bidirectional conditions. Lastly, different weighting schemes were also applied to the phenotypes to reflect the characteristics of real data. Here, MAF based and conservation score based weighting schemes have been considered, and various combination of the above parameters have been used for simulation models. The simulation settings are summarized in Table 1.

**Table 1:** Simulation settings

Index	Simulation settings		
	Weight	Effect model	Conditions
1	No weight	Only interaction effect	Unidirectional
2	No weight	Interaction + marginal effect	Unidirectional
3	No weight	Only interaction effect	Bidirectional
4	MAF weight	Only interaction effect	Unidirectional
5	CONS weight (0.5)	Only interaction effect	Unidirectional
6	CONS weight (0.8)	Only interaction effect	Unidirectional
7	CONS weight (1.0)	Only interaction effect	Unidirectional
8	CONS weight (0.5)	Interaction + marginal effect	Unidirectional
9	CONS weight (0.75)	Interaction + marginal effect	Unidirectional
10	CONS weight (1.0)	Interaction + marginal effect	Unidirectional

**Fig. 5:**
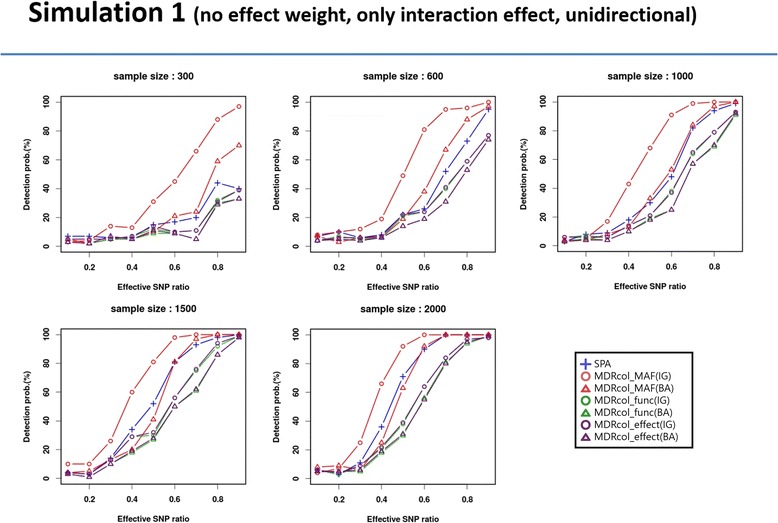
Result of simulation 1

We considered ten simulation settings for this study and evaluated the power. Power is defined as a proportion of causal genes detection among 100 replicates for five different sample sizes (300, 600, 1000, 1500, and 2000) with 10 effective SNP ratios (0.1~ 1.0) on each simulation setting. Among the results of 10 simulation settings, we represented the results of simulation 1 in Fig. 5.

In Fig. 5, the y-axis represents the power of each method and the x-axis represents the effective SNP ratio. In the legend of Fig. 5, ‘SPA’ is referring to the SPA method, ‘MDRcol_’ to our proposed method, ‘MAF’ to MAF-based collapsing, ‘func’ to functional region-based collapsing and ‘effect’ to effect-based collapsing. ‘IG’ and ‘BA’ in parentheses mean information gain and balanced accuracy, respectively, as evaluation measures. Different methods were tested in the dichotomous trait (case/control). As expected, powers of all methods show that patterns increase as sample size and effect SNP ratio increase. These trends are consistently shown in the results of the other simulations. In terms of comparison among collapsing methods, MAF-based collapsing scheme shows higher power than other collapsing schemes. IG shows higher power than BA on same collapsing schemes. These trends are shown in some results of the other simulations, but powers of different collapsing methods and evaluation measures are similar at the same sample size and effective SNP ratio. The effective SNP ratio is defined as the proportion of non-zero weighted SNPs in a region. SPA shows lower power than that of MAF-based collapsing with information gain (‘MDRcol_MAF(IG)’ in Fig. 5) and similar with the other methods. Overall, MAF-based collapsing with information gain (‘MDRcol_MAF(IG)’ in Fig. 5) show the highest power in many cases and similar power with the others in remained cases. GxGrare outperformed SKAT and SPA in the simulations without weight or with MAF weight. In the simulations with conservation weight, GxGrare showed similar performance with SKAT and SPA (Additional file 1: Figure S1-S9).

The type I error, the incorrect rejection of a true null hypothesis, has been measured with 1000 repeats and at the 5% significance level. In the results, all testing methods show about 5% (4.4%~ 5.5%) type I error rates. We observed that the type I error has been well controlled.

### Application to real data (T2D)

We employed a partial set from a study with ~ 13 K individuals from multiple ancestries as part of five whole-exome sequencing studies: the Type 2 Diabetes Genetic Exploration by Next-generation sequencing in multi-Ethnic Samples (T2D-GENES) study. Here, we utilized the Korean subjects in the project, which consists of 1072 individuals and 488,457 autosomal variants.

After quality control with the Hardy-Weinberg equilibrium test and filtering for missing ratio of greater than 0.05 and for MAF of less than 0.01 (rare variants), 414,193 (84.8%) variants remained for the analysis. Gene-based results exhibit no significant genes to be found in relation to T2D using this subset. For adjusting of confounding effects of experimental variables, we use age, sex, BMI, and recruitment area as covariates because of two reasons; Age, sex, and BMI are well-known factors that have association with T2D and two recruitment areas (Ansan and Ansung cohorts) are involved in the study design.

We applied the proposed method with optional parameters and summarized the results with MAF-based collapsing to prevent confusions with different parameters. From the analysis of real data, we investigated gene-gene interaction association between T2D and collapsed rare variants. We report the top 10 gene pairs and their characteristics in Table 2. In Table 2, WCVC is the weighted CVC, BA train is the training balanced accuracy, and BA test is the testing balanced accuracy.

**Table 2:** Top 10 gene combinations in the result

Rank	Gene combination	WCVC	BA train	BA test	*p-value
1	ATP9A, DNAH17	9.924	0.572	0.572	5.00E-06
2	DNAH17, KARS	9.899	0.571	0.571	8.00E-06
3	CHD5, PALB2	9.893	0.571	0.570	1.00E-06
4	DNAH17, EPHB1	9.861	0.569	0.569	1.10E-05
5	ARHGEF16, CHD5	9.855	0.568	0.568	1.00E-06
6	DNAH17, TRPM8	9.848	0.568	0.568	1.70E-06
7	DNAH17, PTPRH,	9.844	0.568	0.568	1.40E-05
8	CARD10, CHD5	9.834	0.567	0.567	5.00E-06
9	CHD5, FAM149A	9.820	0.566	0.566	2.00E-06
10	CHD5, TRANK1	9.814	0.566	0.566	5.00E-06

*p-value is calculated by 1,000,000 permutations

**Fig. 6:**
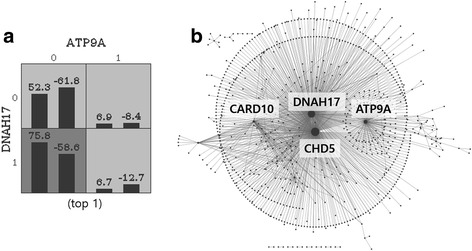
Visualized interaction (**a**) and network diagrams of interactions (**b**)

In Table 2, the values of WCVC are almost 10, which means that gene combinations are selected in almost 10 CV datasets. In other words, gene pairs in Table 2 may not be randomly selected. Additionally, BA train and test are almost the same and this implies that gene pairs may have association with T2D. Among top 10 interactions identified by GxGrare, SKAT identified ARHGEF16-CHD5 (*p* = 0.0301), CARD10-CHD5 (*p* = 0.0009), CHD5-FAM149A (*p* = 0.0391), ATP9A-CHD5 (*p* = 0.0064), CHD5-MLL5 (*p* = 0.0135), while SPA detected no interactions. ATP9A-DNAH17, DNAH17-KARS, CHD5-PALB2, DNAH17-EPHB1, DNAH17-TRPM8, DNAH17-PTPRH were only identified by GxGrare. Among gene pairs, some genes such as CHD5 and DNAH17 have repeatedly appeared. One possible reason is that these genes have a marginal effect. For examples of advanced analysis, we visualized the interaction of top 1 gene pair and network diagram of interactions in Fig. 6.

In Fig. 6 (a), the dark gray cell represents high risk (H) and light gray cells represent low risk (L) in GMDR. One possible explanation for this interaction pattern is that mutations of rare variants in DNAH17 increase T2D risk, but their effects are restricted by the mutations of rare variants in the ATP9A gene. In Fig. 6 (b), each node represents a single gene and the edge represents the association between gene pairs and T2D. In many network diagrams in biology and other domains, hub nodes may have an important role in the property applied in the network construction. In our case, the genes such as CHD5, DNAH17, ATP9A, and CARD10 may have an important role in the occurrence of T2D. These genes may have marginal effects, but the existence of marginal effect does not mean no interaction effects. For example, ATP9A and DNAH17 show the interaction pattern in Fig. 6 (a). A more advanced analysis is possible using the network analysis methods.

Gene-gene interactions in this experiment would represent the simultaneous occurrence of allelic variants in diseased individuals or masking effect of a gene by another. If more diseased individuals have a certain pair of genetic variants, that pair is likely to have gene-gene interaction associated with the disease of interest. Unfortunately, the detected pairs had no reported biological pathways to support the interaction between the genes.

Among the candidate hub genes, CACNA1H, CARD10, FGFRL1, CADPS, ANK2, and MADD have literatures that support their association with T2D; these genes have marginal association with the disease. Although not detected as hubs, TRPM8, PALB2, AXIN1, and CUBN genes have supporting literatures that confirm their association with T2D. These genes have been summarized in Table 3. Other genes on the list can be interpreted as candidate genes with gene-gene interaction without single gene based effects, since these genes have not been detected by single gene based analysis.

**Table 3:** Reported relations between genes and T2D

Gene (region)	Relation to T2D
CACNA1H (coding)	Voltage-Dependent T-Type Calcium Channel Subunit Alpha-1H has a role in Type-1 diabetes [36].
CARD10 (intronic)	CARD10 is a family member that interacts with BCL10 and activates NF-kappa B [37]. Suppression of NF-kappaB activation blocks osteoclastic bone resorption during estrogen deficiency [38], and osteoporosis stems from an imbalance in osteoclastic bone resorption with respect to osteoblastic bone formation [39].
FGRL1 (coding)	Fibroblast Growth Factor Receptor-L1 expression in Pancreatic beta-cells has numerous reports in relation to T2D [40].
CADPS (coding)	This gene encodes a novel neural/endocrine-specific cytosolic and peripheral membrane protein required for the Ca2 + −regulated exocytosis of secretory vesicles [41]. The gene is known to be associated with Type-1 diabetes [42].
MADD (coding)	Tumor necrosis factor alpha is a signaling molecule that interacts with one of two receptors on cells targeted for apoptosis [43]. The gene is known to play a critical role in glucose-induced insulin secretion [44].
ANK2 (coding)	This gene encodes a member of the ankyrin family of proteins that link the integral membrane proteins to the underlying spectrin-actin cytoskeleton. The gene has been reported to be in relation with insulin and pancreatic islets in type-1 diabetes database [45, 46].
TRPM8 (coding)	Mice lacking TRPM8 respond normally to a glucose challenge while exhibiting prolonged hypoglycemia in response to insulin [47]. Relationship between brown adipose tissue, TRPM8 gene, and obesity & diabetes have been reported [48].
PALB2 (coding)	This gene encodes a protein that may function in tumor suppression [49]. This gene is reported to be related to Breast cancer and pancreatic cancer [50]. Pancreatic cancer and diabetes have close relations [51].
AXIN1 (coding)	This gene encodes a cytoplasmic protein which contains a regulation of G-protein signaling domain and a disheveled axin domain [52]. The genes is reported in the type-1 diabetes database.
CUBN (coding)	Cubilin (CUBN) acts as a receptor for intrinsic factor-vitamin B12 complexes [53]. The gene is related to albuminuria and is an important key factor of chronic kidney disease, especially in individuals with diabetes [54].

## Discussion and conclusion

In this paper, we propose a weight based collapsing framework for rare variants association analysis that investigates gene-gene interaction. Three collapsing schemes are suggested through common weighting mechanism for each rare variant. Of the two key findings, first is that the MAF based collapsing method showed the best overall performance in the results of simulation data, in terms of power (while preserving type I error), in our simulation studies comparing SPA, SKAT and the three proposed collapsing methods explained in detail in our methods section. This result was somewhat surprising in that the simple collapsing of variants with similar MAFs were able to find more candidate interactions than other methods; other than the SPA, the other two collapsing methods contain more biological information that was expected to be advantageous, however, was shown otherwise. The second finding is that using the IG as evaluation measure showed higher statistical power than using the BA as evaluation measure, when using our MDR scheme. Many have coupled BA with MDR analyses to report top interactions and biological validations have been performed for such interactions. Since using the IG performed better than using BA in terms of power, while preserving type I error, when analyzing our simulation datasets, we expect our findings with the real data to be plausible candidates for early gene-gene interactions. Entropy measures such as IG and normalized mutual information (NMI) have shown better performance than the BA to measure the classification performance in the contingency table [21]. As compared with BA, these entropy measures are less susceptible to over-fitting and superior to classification error [34, 35]. As stated in the results section, some of the findings were already reported to have associations with the disease of interest, and the rest of the genes in the list should be investigated through biological validation methods.

Our study presents a novel scheme to analyze the gene-gene interaction of rare variants. Several weight collapsing schemes have been compared and incorporation of IG as evaluation measure has been added to MDR analysis. We identified several interacted gene pairs and hub-genes that are related to the disease of interest. Although the current study compared three types of proposed collapsing schemes and suggested one evaluation measure other than BA, our approach illustrates examples of a practical way to improve existing methods. In further studies, a universally optimal weight collapsing scheme will be investigated. As public biological databases accumulate, functional information and conservation score information will be updated; improved performances of collapsing methods using biological databases can be assumed. Moreover, our framework can be easily expanded to utilize other types of future annotations and to compare their performances as MDR weights.

GxGrare is available at http://bibs.snu.ac.kr/software/gxgrare which contains simulation data and documentation. Supported operating systems include Linux and OS X.

## Additional file

Additional file 1: Figure S1–S9, detection probabilities of simulation 2~ 10. (DOCX 706 kb)
