# Supplementary material for: GxGrare: gene-gene interaction analysis method for rare variants from high-throughput sequencing data
Source: BMC Syst Biol. 2018 Mar 19;12(Suppl 2):19. doi: 10.1186/s12918-018-0543-4 (PMC5861485; doi:10.1186/s12918-018-0543-4)

Additional file 1

Figure S1. Detection probability of simulation 2


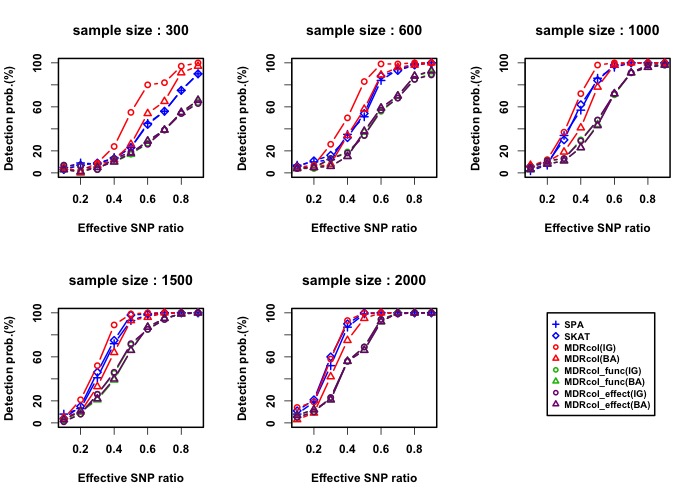


Figure S2. Detection probability of simulation 3


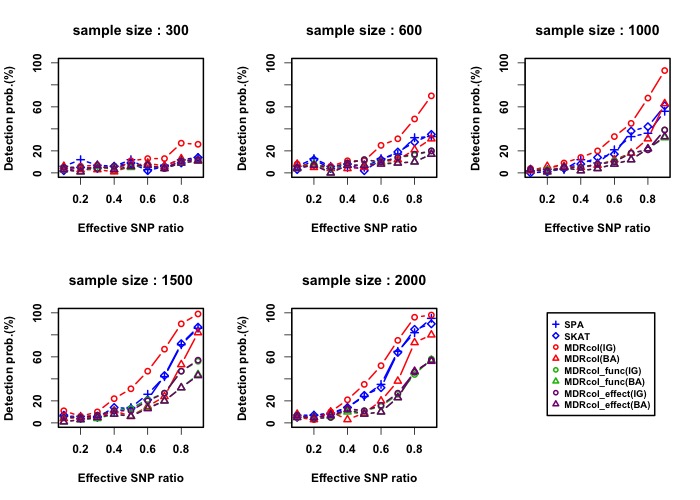


Figure S3. Detection probability of simulation 4


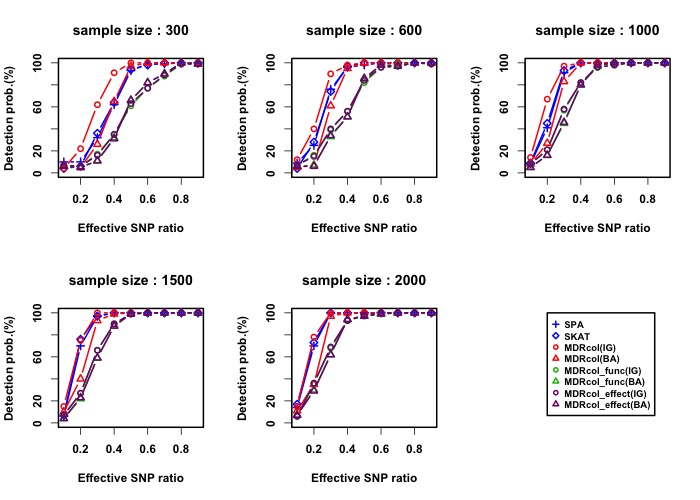


Figure S4. Detection probability of simulation 5


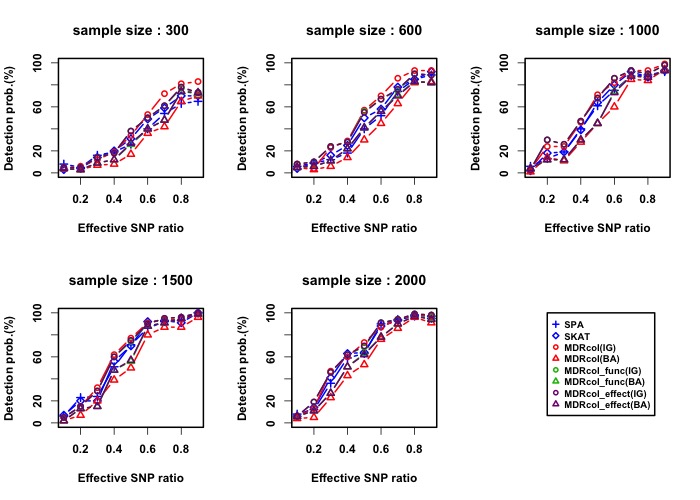


Figure S5. Detection probability of simulation 6


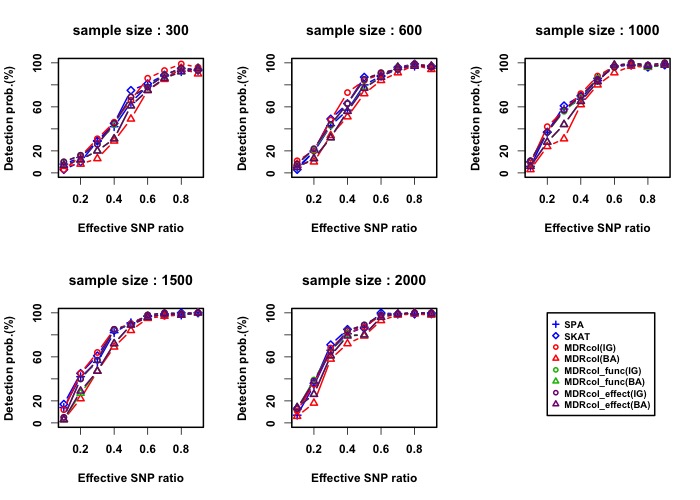


Figure S6. Detection probability of simulation 7


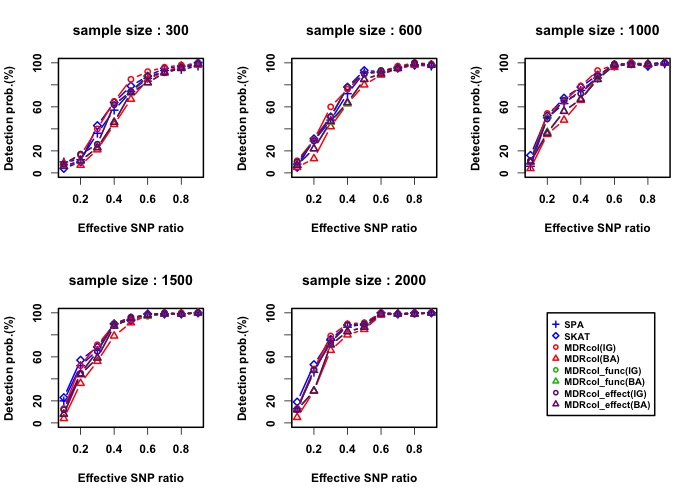


Figure S7. Detection probability of simulation 8


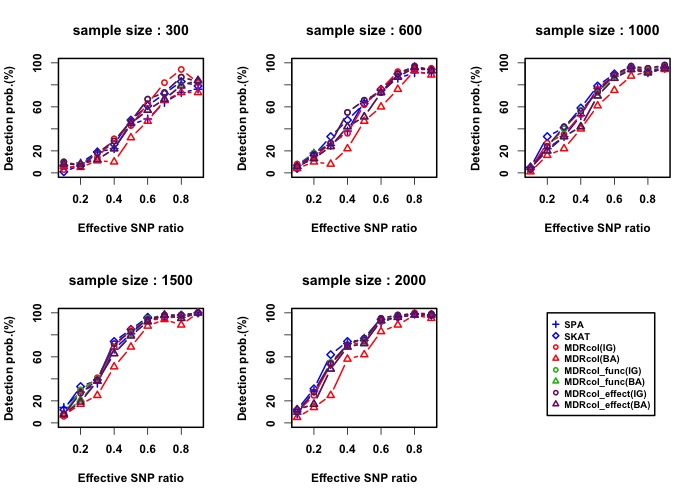


Figure S8. Detection probability of simulation 9


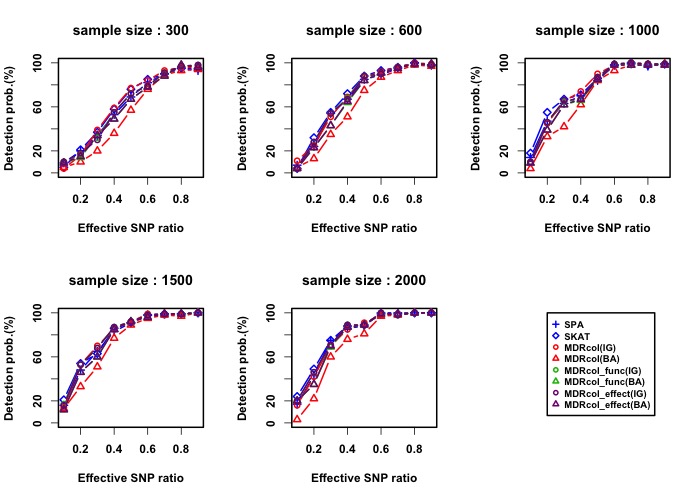


Figure S9. Detection probability of simulation 10


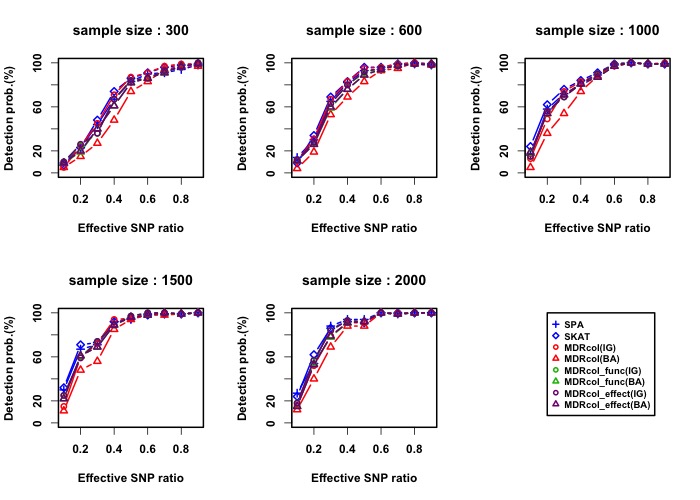

Supplement: Supplementary file 1 — Figure S1–S9, detection probabilities of simulation 2~ 10. (DOCX 706 kb) [file 12918_2018_543_MOESM1_ESM.docx]
